# Auricular acupressure as a complementary therapy for psoriasis vulgaris: study protocol for a multicenter randomized controlled trial

**DOI:** 10.1186/s13063-019-3475-4

**Published:** 2019-06-17

**Authors:** Jingwen Deng, Chuanjian Lu, Yu Xiang, Hao Deng, Zehuai Wen, Danni Yao, Meiling Xuan, Yuhong Yan

**Affiliations:** 1 Psoriasis Clinical and Basic Research Team, Guangdong Provincial Hospital of Chinese Medicine, Guangzhou, 510120 China; 2 Guangdong Provincial Academy of Chinese Medical Sciences, Guangzhou, 510120 China; 3 Guangdong Provincial Key Laboratory of Clinical Research on Traditional Chinese Medicine Syndrome, Guangzhou, 510120 China; 4 Key Unit of Methodology in Clinical Research, Guangdong Provincial Hospital of Chinese Medicine, Guangzhou, 510120 China; 5 School of Medical Information Engineering, Guangdong Pharmaceutical University, Guangzhou, 510006 China

## Abstract

**Introduction:**

Psoriasis vulgaris is a common skin disease characterized by persistent localized erythematous scaly plaques, typically on the elbows, knees, and scalp. It is an immune-abnormal disease that progresses slowly over a long period with frequent symptom recurrence. Current studies have shown that acupuncture is an effective therapy for psoriasis. However, the scientific evidence of the efficacy of auricular acupressure treatment for patients with psoriasis is still insufficient. Therefore, we designed a randomized controlled clinical trial to investigate the effect, safety, and cost-effectiveness of auricular acupressure in addition to medication in patients with psoriasis.

**Methods and analysis:**

This on-going study is a two-arm parallel, assessor-blinded, randomized controlled trial in which 180 participants with psoriasis will be recruited and then randomly allocated into two groups in a 1:1 ratio. Equal randomization will be conducted using a computer-generated random allocation sequence. Participants in the intervention group will receive auricular acupressure treatment once per week for 4 weeks, and calcipotriol betamethasone ointment for topical use once daily for 4 weeks. Participants in the control group will receive only calcipotriol betamethasone ointment treatment once daily for 4 weeks. All patients will be followed up for 12 weeks. The primary outcome is relapse rate. The secondary outcomes include time to relapse, rebound rate, time to new onset, Psoriasis Area and Severity Index score improvement rate, body surface area affected, a visual analogue scale, and Dermatology Life Quality Index. Cost-effectiveness analysis will be carried out from a health and community care provider perspective.

**Discussion:**

This multicenter randomized controlled trial will provide important clinical evidence for the effect and safety of auricular acupressure as a complementary therapy in patients with psoriasis.

**Trial registration:**

Chinese Clinical Trial Registry, ChiCTR-TRC-14004916. Registered on 20 May 2014. This protocol is version 3.0 which was updated on 24 September 2016.

**Electronic supplementary material:**

The online version of this article (10.1186/s13063-019-3475-4) contains supplementary material, which is available to authorized users.

## Background

Psoriasis vulgaris is an immune-abnormal, chronic skin disease characterized by well-delineated red, scaly plaques. It is induced by a number of environmental factors and arouses great public concern because of its high prevalence, impact on life quality, and incurable characteristics [1]. With a prevalence of 1% to 3%, psoriasis is likely to be encountered by general practitioners [2]. Furthermore, psoriasis has also been associated with a significantly increased risk of myocardial infarction, stroke, and peripheral vascular disease, possibly because of accelerated atherosclerosis in the setting of an inflammatory state [3].

Traditional Chinese medicine (TCM) is based on the fundamental principle of Yin-Yang balance, Five Elements, and a relationship between humans and nature [4]. When treating diseases, TCM first evaluates presenting symptoms to differentiate the syndrome related to the disease and then clarifies a therapeutic method for this disease. For more than 2000 years, TCM has been used to treat various diseases in China and throughout East Asia, and it still remains the first choice of treatment for many people because its effectiveness and its cost being inexpensive. Clinical practices have also proved that TCM is beneficial and effective in alleviating clinical symptoms, improving quality of life, immune function, reducing metastasis, and preventing recurrence in various diseases [5–10].

In the perspective of TCM, the pathogenesis of psoriasis is deficiency in origin and excess in symptom (Ben Xu Biao Shi), and in most cases it co-occurs with blood stasis. In chronic psoriasis, *qi* was exhausted during a long disease course and then the blood circulation was disturbed. Finally, *qi* and blood coagulated and blocked the meridians, which led to lack of nourishment for the skin and muscle. Thus, TCM clinical experts advocated ‘treatment of psoriasis from the blood aspect’ [11, 12]. The effective ways to achieve this are blood-activating herbal medicines and treatments such as acupuncture and bloodletting. A systematic review indicated that the evidence for the effect of multi-herb formulations for psoriasis was promising in a number of the studies [13]. A meta-analysis reported that Chinese medicine was effective for psoriasis vulgaris since it is noninferior to acitretin and it could produce add-on effects when combined with acitretin, as well as reducing the acitretin-induced adverse events [14]. Chinese medicine combined with narrowband ultraviolet B for treating psoriasis also showed improved efficacy [15, 16]. Herbal formulae or plant extract topical management were analyzed in other systematic reviews, which showed that topical herbal formulae or plant extracts could improve overall clinical symptoms [17–19].

**Fig. 1:**
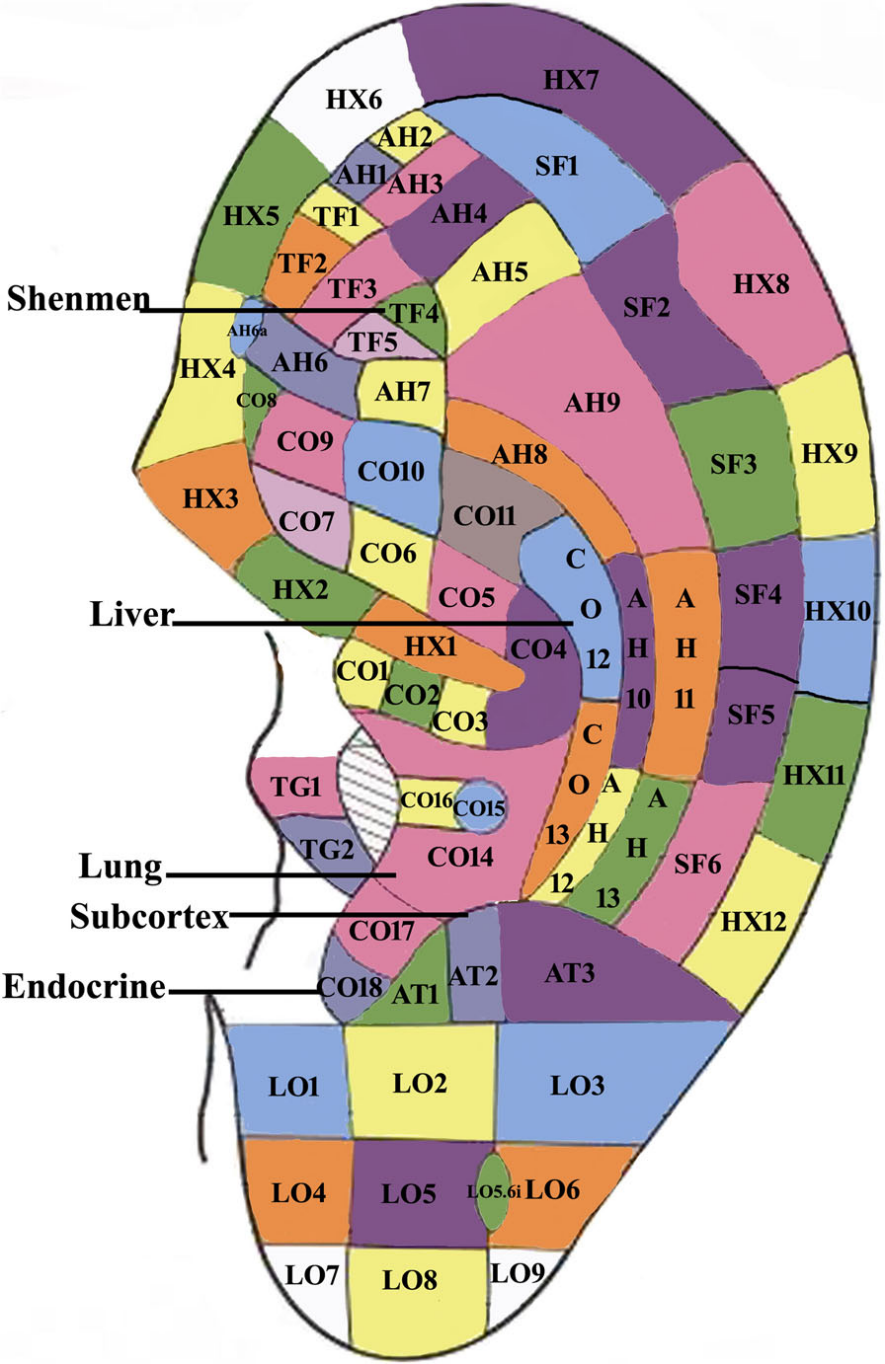
Standard codes for the division of the auricle

Acupuncture is a typical representative of TCM, and is one of the various general therapies for psoriasis. In a systematic review, acupuncture therapies show some evidence of benefit for the treatment of psoriasis [20]. Auricular acupressure is the acupuncture-related practice. It is a noninvasive form of acupuncture which uses physical pressure applied to the auricular acupressure point (AAP) by *Vaccaria* seeds [21]. The prescription component of auricular acupressure is principally based on the divisions of the auricle, with definite terms and denoting s [22]. Named AAPs and regions are distributed over the entire surface of the auricle (Fig. 1).

Auricular acupressure is characterized by balancing Yin and Yang, dredging meridians and collaterals, regulating the functions of organs, and building up body resistance to pathogenic factors [23]. Auricular acupressure has been used to treat various diseases. In recent studies, auricular acupressure has been shown to help menstruating women effectively relieve menstrual headache [24]. Furthermore, it has been shown that auricular acupressure treatment can improve sleep quality and daytime dysfunction in insomnia patients [25–27]. By stimulating a point on the ear, a promising therapeutic effect is believed to occur on the gross anatomical organ associated with that point. Studies have demonstrated that auricular therapy can promote general *qi* and blood circulation; hence, it can eventually improve the blood flow [28].

Since there is little experience using auricular acupressure in psoriasis, we aim to analyze the additional efficacy on symptom improvement and relapse rate associated with auricular acupressure treatment for psoriasis. The aim of our study is to show whether there is additional benefit in improving symptoms and preventing recurrence when adding auricular acupressure treatments to usual therapy compared with usual therapy alone for people with psoriasis.

## Methods/design

### Study design

This study is a two-parallel arm, assessor-blinded, randomized controlled trial. The trial will be conducted by Guangdong Provincial Hospital of Chinese Medicine (GPHCM) and performed at two clinical research centers in China (Guangdong Provincial Hospital of Chinese Medicine and Guangzhou Red Cross Hospital) in accordance with the Declaration of Helsinki and the Guidelines for Good Clinical Practice.

**Fig. 2:**
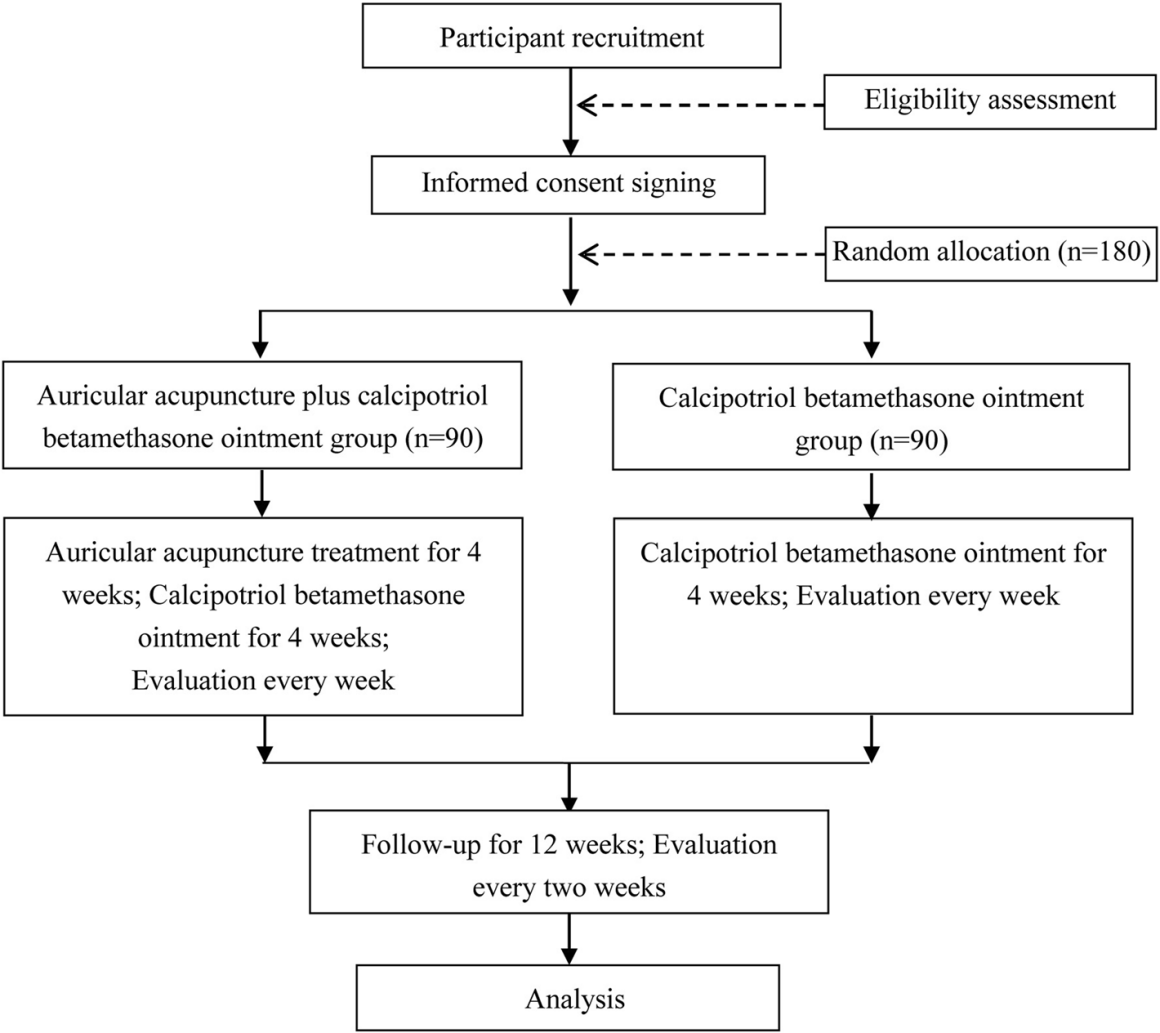
Flowchart of the study

This study will select patients with blood stasis syndrome on a TCM diagnosis based on a TCM syndrome differentiation standard [29, 30]. Eligible participants will be stratified by age and sex and randomly allocated in a 1:1 ratio to one of the treatment arms (the auricular acupressure plus calcipotriol betamethasone ointment group or the control calcipotriol betamethasone ointment group) and receive treatment for 4 weeks, with 12 weeks of follow-up (Fig. 2). The Standard Protocol Items: Recommendations for Interventional Trials (SPIRIT) checklist is provided in Additional file 1.

### Participants

#### Inclusion criteria

A total of 180 patients will be recruited from the outpatient clinics of the two hospitals. The eligibility criteria for the study include: 1) those with a diagnosis of psoriasis vulgaris (referring to the 2008 clinical guidelines of psoriasis reported by the Chinese Medical Association [31]; 2) aged 18 to 70 years; 3) psoriasis vulgaris patients (Psoriasis Area and Severity Index score (PASI) score of 7–20, with body surface area (BSA) affected < 30%); and 4) willing to sign a written informed consent.

#### Exclusion criteria

Participants will be excluded if they are experiencing or have a history of the following: 1) psoriatic arthritis, guttate psoriasis, inverse psoriasis or exclusively involving the face; 2) pregnancy, breast-feeding, or those who intend to become pregnant within 1 year; 3) a Self-rating Anxiety Scale (SAS) > 50 or Self-rating Depression Scale (SDS) > 53, or with other psychiatric disorders; 4) important systemic disease that cannot be controlled through common treatment (either with infection, electrolyte imbalance, acid-base disturbance, calcium metabolic disorder, or cancer; 5) those allergic to any medicine or ingredients used in this study; 6) those participating in other clinical trials or those who have participated within 1 month before entry into the study; 7) those who have been treated with topical treatments (i.e., corticosteroids, retinoic acid) within 2 weeks, or systemic therapy or phototherapy (ultraviolet B) and psoralen combined with ultraviolet A within 4 weeks, or biological therapy within 12 weeks before beginning the study.

### Randomization

Eligible patients will be randomly assigned in a 1:1 ratio to one of the two groups (the treatment group who receive auricular acupressure plus calcipotriol betamethasone ointment or the control group who receive calcipotriol betamethasone ointment) at the second visit through central randomization. Equal randomization will be conducted using a computer-generated random allocation sequence through the stratified block randomization method of the SAS software (version 9.12; SAS Institute, Inc., Cary, NC, USA) with a block size of 4 by the Key Unit of Methodology in Clinical Research (KUMCR) of Guangdong Provincial Hospital of Chinese Medicine. Allocation concealment will be ensured since the randomization code will be released by the Interactive Web Response System for Chinese Medicine Trials (IWRS-CMT), which is a verified online randomization facility established by the KUMCR (http://www.gztcmgcp.net/sjxt /login.asp). Following this, the participants will be randomly allocated to the two different groups. The practitioners will be aware of the allocation arm according to the different medical procedures. However, the evaluation of participants and the analysis of the results will be performed by physician assessors and statisticians who are blinded to the group allocation [32].

### Intervention

Participants in both groups will be provided with calcipotriol betamethasone ointment. Subjects randomized to the experimental group will receive integrated auricular acupressure therapy and calcipotriol betamethasone ointment topical therapy.

### Auricular acupressure treatment

Patients in the auricular acupressure plus calcipotriol betamethasone ointment group will receive auricular acupressure treatment once a week for 4 weeks. At every visit, therapists treat the patients with auricular acupressure. All of the therapists participated in this trial will be standardized in their training, including in the study protocol and methods of treatment. We selected auricular acupoints as suggested in the nomenclature and location of auricular points [33]. The auricular acupoints are based on a set of anatomical maps superimposed onto the ear. The following auricular points will be used for treatment: Lung (Fei, CO14), Shenmen (TF4), endocrine (CO18), Subcortex (AT4) and Liver (Gan, CO12) (Fig. 1). According to modern research and Chinese medicine, ‘The liver (Gan) stores blood. Liver dispersion, *qi* stagnation’, ‘The lung (Fei) governs skin and hair’, and, in addition to treating respiratory-related disease, the auricular points Liver (CO12) and Shenmen (TF4) work on regulating emotion and reducing stress, anxiety, and excessive sensitivity [34, 35]. Lung (CO14) also works on relieving painful and itchy skin diseases [36]. Endocrine (CO18) and Pizhixia (AT4) could be helpful with endocrine hormone balance, hypersensitivity, and rheumatism [37, 38].

After sterilization with 75% alcohol, *Vaccaria* seeds will be stuck and fixed on the above auricular points of both ears, with each pressed individually for 1 min to induce stimulus until patients feel endurable heat and distending pain on the auricles, and patients are advised to repeat the pressing themselves four times daily for 5 consecutive days and then to remove the seeds stuck to their auricles on the morning of day 6, which is helpful to avoid contact dermatitis caused by long-term adhesive tape.

### Calcipotriol betamethasone ointment treatment

Traditional topical therapies (such as corticosteroids, vitamin D and analogues, dithranol, and tar preparations) are recommend by NICE guideline as first-line therapy [39]. In this study, we used a routine drug for first-line therapy in the control group and as a base treatment in the intervention group. Thus, participants in both groups will be provided with calcipotriol betamethasone ointment (Daivobet® gel; calcipotriol 50 μg/g plus betamethasone 0.5 mg/g) for topical use once daily until the PASI score of the patient is reduced to 0. However, the course of calcipotriol betamethasone ointment treatment would last no more than 4 weeks.

### Outcome

#### Primary outcome measurement

The primary outcome measure in the trial is relapse rate in the treatment period and follow-up period. Relapse is defined as losing 50% of the improvement obtained from treatment once the treatment is stopped [40]. The PASI will be assessed every visit during the treatment period and the follow-up period. Meanwhile, patients will be required to report the emergence of a variety of conditions at any time in the study period. Assessors will evaluate the PASI score on the same or closest day. Target lesions will be recorded as digital photographs by single-lens reflex (SLR) cameras at every visit.

#### Secondary outcome measurements

Secondary outcome measures include the time to relapse, rebound rate, time to new onset, PASI improvement rate, BSA affected, a visual analog scale (VAS), and Dermatology Life Quality Index (DLQI). Time to relapse is defined as the time it takes to lose 50% of the improvement obtained from treatment (Table 1). Time to new onset is the PASI score improvement achieved (PASI-50) for the first time, but a failure to keep the PASI-50 improvement all through the treatment period. Rebound is defined as a PASI score of 125% of that at baseline, or the occurrence of new generalized pustular, erythrodermic, or more inflammatory psoriasis occurring [41]. The VAS and BSA will be assessed every week during the first 4 weeks and every 2 weeks in the follow-up period. The DLQI will be self-assessed by patients every 4 weeks throughout the trial. Laboratory reports will be also monitored until the last visit. TCM syndrome is one of the assessments which will be recorded before and after the auricular acupressure, as well as at the end of the follow-up period (Table 2).

**Table 1:** Summary of measurement

Outcome	Measurement tool	Details
Primary outcome		
Relapse rate	Psoriasis Area and Severity Index (PASI) score	Relapse defined as losing 50% of the improvement obtained from treatment once the treatment is stopped
Secondary outcome		
Time to relapse	PASI score	Time domain between achieving at least 50% reduction in PASI score and losing 50% of the improvement obtained from treatment once the treatment is stopped.
Time to new onset	PASI score	Time domain between lesion clearing and recurrence
Rebound rate	PASI score	Rebound defined as a severe and sudden change in the severity of psoriasis that is significantly worse than before the treatment was initiated
PASI improvement rate	PASI score	PASI score is a tool used to measure the severity and extent of psoriasis. It takes a few minutes and experience to calculate it accurately. A representative area of psoriasis is selected for each body region. The intensity of redness, thickness, and scaling of the psoriasis is assessed as none (0), mild (1), moderate (2), severe (3), or very severe (4)
VAS	Visual analog scale (VAS)	VAS is the most common pain scale for quantification of endometriosis-related pain. We use VAS to evaluate the feeling of pruritus in participants during the treatment period
BSA	Body surface area (BSA)	BSA is a common measure in the medical field and part of the complete body size and composition profile
DLQI	Dermatology Life Quality Index (DLQI)	DLQI is a dermatology-specific quality of life instrument. It is a simple 10-question validated questionnaire. At present, the DLQI is the most frequently used instrument in studies of randomized controlled trials in dermatology

**Table 2:**
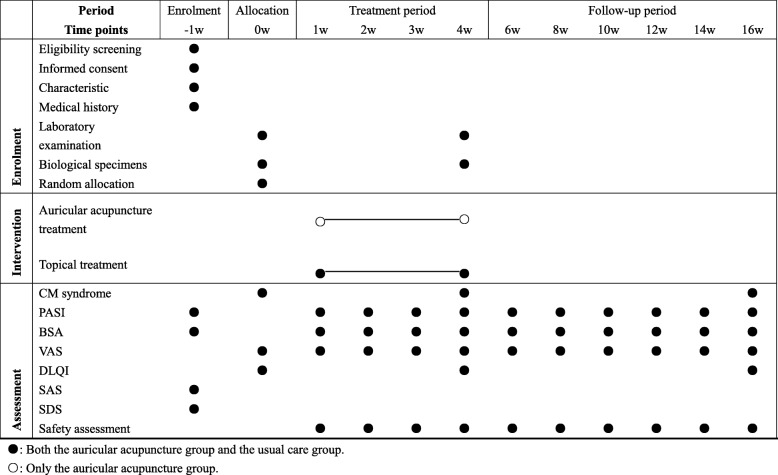
Schedule for treatment and outcome measurements

*BSA* body surface area, *CM* Chinese medicine, *DLQI* Dermatology Life Quality Index, *PASI* Psoriasis Area and Severity Index, *SAS* Self-rating Anxiety Scale, *SDS* Self-rating Depression Scale, *VAS* visual analog scale

### Health economics

Economic evaluation will be carried out from the perspective of the Health Department of Guangdong Province, which will be in the form of a cost-utility analysis conducted using utility values obtained from the DLQI preference-based quality of life measure. DLQI is a dermatology-specific quality of life instrument for routine clinical use. It is a validated questionnaire with 10 simple questions. At present, the DLQI is the most frequently used instrument for evaluating the impact of skin disease and related treatment on the lives of patients. The DLQI will be measured at baseline and at 4 and 16 weeks for utility-based quality of life evaluation in this study. Resource use will include intervention costs, healthcare costs, and community service costs, which will be calculated for each trial participant. We will analyze an incremental cost-effectiveness ratio (ICER) of cost per patient by calculating the incremental mean difference in costs between the two trial arms and incremental difference in patient outcome after the follow-up.

### Sample size

There is a growing body of research evaluating the effect of auricular acupressure for various diseases. However, there is still a lack of studies evaluating the effect of auricular acupressure on psoriasis. There is no previous study on which to base the sample size calculation. Thus, we used the primary outcome relapse rate to determine a sample size calculation. Based on the study of Menter et al. [42], the relapse rate of calcipotriol betamethasone ointment topical therapy after 4 weeks of treatment and 4 weeks of follow-up is 82%. We assume that the relapse rate of auricular acupressure therapy combined with calcipotriol betamethasone ointment topical therapy for psoriasis in week 12 is 20% less than topical therapy alone and that the relapse rate of calcipotriol betamethasone ointment topical therapy is 82%. On the basis of this hypothesis, and in order to compare the two groups for a significance level α = 0.05 and a power 1 – β = 0.80, we calculated the sample size by PASS statistics software (version 11.0.10; NCSS LLC., Utah, USA); 77 patients with psoriasis would have been required in each group to achieve 80% power to detect a difference between the group proportions of −0.2. The test statistic used is the two-sided *Z* test with pooled variance. The significance level of the test is targeted at 0.05. Considering 15% loss to follow-up, 90 patients are needed in each arm, totaling 180 patients in all.

### Data collection

Outcome measurements will be carried out and recorded using paper case report forms (CRF) and checked for each participant at every visit by a certificated clinical researcher. To promote patient enrolment, retention, and completion of follow-up, all the treatments and laboratory tests will be free. A 300 RMB gift will be sent to participants when they have completed the follow-up.

### Data management

Data managers will enter the information on the CRF into an electronic database using the double-entry method. There is no exclusive trial steering committee named in this trial. The integrity of CRF data in different centers will be monitored regularly by Guangdong International Clinical Research Center of Chinese Medicine (Guangzhou, China). As well as the safety data, and the critical efficacy outcomes will be assessed by the Data Monitoring Committee from GPHCM.

Patients will be pseudonymized by study identification numbers for participant confidentiality. Access to the CRF database will be limited to the study group and the coordinator.

### Statistical analysis

All analyses will be performed with PASS Statistics and SAS 9.2 software by a statistician who is blinded to the random allocation of groups. An intent-to-treat (ITT) basis statistical analysis with a 95% confidence interval will be performed using multiple imputations. The major imputation is multiple imputation by chained equations (MICE). The ITT analysis will include all patients who are randomized [43]. Safety analysis will be undertaken by analyzing the frequency of adverse events which are suspected as related to the treatment. Planned subgroup analyses include those based on the type of TCM syndromes and severity of psoriasis. The various parameters observed will be compared using a Chi-square test for noncontinuous variables (i.e., the primary outcome relapse rate) and *t* test and analysis of variance (ANOVA) for continuous variables. In order to distinguish the treatment effect and time effect, the repeated measures ANOVA change from baseline will be performed for the different time point assessments. Statistical significance is established at *P* < 0.05. No formal interim analysis is planned.

### Adverse events

Before the start of treatment and after 4 weeks of treatment a medical history will be recorded for each patient, and standard laboratory examinations and specific laboratory investigations will also be performed. The standard laboratory examinations include: hematologic parameter assessments (hemoglobin, red blood cells, platelets, and white blood cell counts); urinalysis (proteins, red blood cell and white blood cell biochemical assessment (serum electrolytes)); indices of renal function (creatinine, urea) and hepatic function (alkaline phosphatase, aspartate aminotransferase, alanine aminotransferase, and gamma-glutamyl transpeptidase); serum calcium; and an electrocardiogram. The specific laboratory investigations mainly include serum cytokine levels.

All adverse events will be collected and graded for severity and potential relation to the treatments in the study by assessors at every visit. Safety evaluations include the incidence of treatment-induced or serious adverse events, dropout because of adverse events, and changes from baseline of the PASI score and laboratory parameters. Patients will be also asked about other adverse effects of auricular therapy such as faintness, nausea, and vomiting. In case of severe adverse effects, auricular therapy will be discontinued immediately.

## Discussion

Psoriasis is a chronic inflammatory disease that manifests as a wide spectrum of clinical signs ranging from variable skin symptoms to arthritis. Effective treatments are available for the routine care of individuals with psoriasis. Various therapeutic regimens including different topical corticosteroids, topical vitamin D analogs, biological agents, phototherapy, photochemotherapy, cyclosporin, systemic therapy with methotrexate, and combination therapies have shown beneficial therapeutic effects for patients with psoriasis [44]. However, some of these treatments are expensive, some require appropriate monitoring, and some may only be accessed in clinical care settings because of potential adverse events. However, with there being no cure, the aim of therapies for people with psoriasis is to minimize the extent and severity of the disease so that it no longer substantially impacts their quality of life. Evidence indicates that, currently, a substantial proportion of patients are dissatisfied with their treatment for psoriasis. Besides, patients who discontinue treatments may experience a return of the disease or worsening of the disease [45]. This detrimental impact on quality of life is a relentless condition yet one for which many people have given up seeking any medical support.

Hence, relapses are common in psoriasis and patients may have to maintain therapy for long durations. However, the pattern of relapse varies. Some patients have early and frequent relapses and others may have long-term remissions with infrequent relapses [46]. In the study by Kaur et al. the duration of remissions varied widely from 2 weeks to 9 years, and 4% of patients never had complete remission in India [47]. Studies on cyclosporine in psoriasis found that 50–60% of patients relapsed 6 months after treatment withdrawal and that the time to relapse depended on the severity of disease, the dose required to achieve clearance, and the extent of clearing achieved before termination of the drug [48]. In the study by Heydendael et al. PASI-75 was achieved in 60% of patients with cyclosporine treatment, but discontinuation often led to a relapse [49].

Consequently, to find more effective therapeutic methods to prevent the return of disease activity many individuals have turned their attention to treatments such as TCM. Currently, clinical experience has been accumulated for TCM treatment of psoriasis [13–19, 50–52]. Most significantly, as a type of TCM, acupuncture has shown respectable efficacy and is broadly accepted internationally.

Auricular acupressure is defined as a healthcare modality whereby the external surface of the ear, or auricle, is stimulated to alleviate pathological conditions in other parts of the body [52]. The auricular acupoints are based on a set of anatomical maps superimposed onto the ear. The stimulation of auricular acupoints is intended to regulate *qi*, activate the meridians, and is proposed to affect the gross anatomical organ associated with that point. In so doing, a variety of health problems (for example, chronic pain, insomnia, and lactation disorder) have been successfully cured [53–56]. Systematic reviews of acupuncture therapy for psoriasis indicated that acupuncture-related techniques could be considered as an alternative or adjuvant therapy for psoriasis [20, 57, 58]. We found some evidence of a benefit of auricular acupressure for the treatment of psoriasis vulgaris [59, 60]. However, the conclusions are limited by the small number of included trials from single studies. We aim to clarify the effect of auricular acupressure for psoriasis but are unable to find any previous published studies that addressed this question. Thus, the present study is designed to look at the relapse rate in psoriasis with auricular acupressure plus usual treatment after achieving PASI-50, and it may contribute to this aim when auricular acupressure is used in addition to medication in the present design.

The primary aim of the present study is to evaluate the control of psoriasis recurrence after auricular acupressure plus usual treatment in patients. The secondary aim is to evaluate the effect of the auricular acupressure plus usual treatment versus usual treatment alone on cutaneous symptom reduction at 4 weeks. We hypothesize that the combined treatment group will contain a higher proportion of patients who are better maintaining their recover status after achieving PASI-50, and who have a greater reduction in cutaneous symptoms at 4 weeks, compared with the usual treatment group. We are unable to find any previously published studies that addressed these questions. This study is one of the first trials to evaluate the effect of auricular acupressure on psoriasis. It may contribute to the aims described above when auricular acupressure is used in addition to medication.

## Trial status

The recruitment phase began in March 2013. Thus far, 154 patients have been recruited.

## Additional file

Additional file 1: SPIRIT 2013 checklist: recommended items to address in a clinical trial protocol and related documents. (DOC 126 kb)

## Data Availability

Not applicable.

## Abbreviations

AAP: Auricular acupressure point
ANOVA: Analysis of variance
BSA: Body surface area
CRF: Case report forms
DLQI: Dermatology Life Quality Index
GPHCM: Guangdong Provincial Hospital of Chinese Medicine
ICER: Incremental cost-effectiveness ratio
ITT: Intent-to-treat
IWRS-CMT: Interactive Web Response System for Chinese Medicine Trials
KUMCR: Key Unit of Methodology in Clinical Research
MICE: Multiple imputation by chained equations
PASI: Psoriasis Area and Severity Index
SAS: Self-rating Anxiety Scale
SDS: Self-rating Depression Scale
TCM: Traditional Chinese medicine
VAS: Visual analog scale
